# Vestibular modulation of the tail of the rat striatum

**DOI:** 10.1038/s41598-023-31289-1

**Published:** 2023-03-17

**Authors:** Faezeh Tashakori- Sabzevar, Nico Vautrelle, Yiwen Zheng, Paul F. Smith

**Affiliations:** 1 Department of Pharmacology and Toxicology, University of Otago, Dunedin, New Zealand; 2 Department of Anatomy, School of Biomedical Sciences, and Brain Health Research Centre, University of Otago, Dunedin, New Zealand; 3 The Eisdell Moore Centre for Hearing and Balance Research, University of Auckland, Auckland, New Zealand

**Keywords:** Neuroscience, Motor control, Basal ganglia, Physiology, Neurophysiology

## Abstract

Fragmented and piecemeal evidence from animal and human studies suggests that vestibular information is transmitted to the striatum, a part of the basal ganglia that degenerates in Parkinson’s Disease. Nonetheless, surprisingly little is known about the precise effects of activation of the vestibular system on the striatum. Electrophysiological studies have yielded inconsistent results, with many studies reporting only sparse responses to vestibular stimulation in the dorsomedial striatum. In this study, we sought to elucidate the effects of electrical stimulation of the peripheral vestibular system on electrophysiological responses in the tail of the rat striatum, a newly discovered region for sensory input. Rats were anaesthetised with urethane and a bipolar stimulating electrode was placed in the round window in order to activate the peripheral vestibular system. A recording electrode was positioned in the tail of the striatum. Local field potentials (LFPs) were recorded ipsilaterally and contralaterally to the stimulation using a range of current parameters. In order to confirm that the vestibular system was activated, video-oculography was used to monitor vestibular nystagmus. At current amplitudes that evoked vestibular nystagmus, clear triphasic LFPs were evoked in the bilateral tail of the striatum, with the first phase of the waveform exhibiting latencies of less than 22 ms. The LFP amplitude increased with increasing current amplitude (P ≤ 0.0001). In order to exclude the possibility that the LFPs were evoked by the activation of the auditory system, the cochlea was surgically lesioned in some animals. In these animals the LFPs persisted despite the cochlear lesions, which were verified histologically. Overall, the results obtained suggest that there are vestibular projections to the tail of the striatum, which could possibly arise from projections via the vestibular nucleus or cerebellum and the parafasicular nucleus of the thalamus.

## Introduction

The vestibular system is a sensory system that encodes angular and linear acceleration of the head in 3 dimensions and has a pivotal role in generating eye movement to prevent retinal slip and stabilize gaze (i.e., the vestibulo-ocular reflexes), and in regulating posture and locomotion (i.e., the vestibulo-spinal reflexes). Vestibular information about head motion and position is integrated with corresponding information from other sensory inputs such as vision, hearing, proprioception and olfaction, in many areas of the brain, in order to provide a stable estimate of where we are in the environment. Therefore, loss of normal vestibular function results in a syndrome of ocular motor and postural symptoms, which include abnormal vestibulo-ocular reflexes, abnormal vestibulo-spinal reflexes manifesting in postural instability and abnormal locomotion, as well as dizziness and vertigo (see Ref.^1^ for a review).

For decades, it has been speculated that the vestibular system may have connections with the basal ganglia, due to the importance of areas like the striatum in voluntary motor control. The basal ganglia play a fundamental role in the planning and execution of movements, decision making, and reward-mediated learning^2^. It is well demonstrated that the basal ganglia play an important role in the control of saccadic eye movement [e.g., Ref.^3^; see Ref.^4^ for a review]. Basal ganglia dysfunction in Parkinson's disease (PD) causes significant tremor, rigidity, bradykinesia, postural instability as well as impaired locomotion^5,6^. However, potential neural pathways between the vestibular system and the striatum have been a matter of controversy. For example, evidence has been presented for a pathway between the medial vestibular nucleus and the dorsolateral putamen of the striatum via the parafasicular nucleus (PFN)^7^. However, other studies have suggested that vestibular information may be transmitted to the dorsal striatum (i.e., the caudate nucleus and the putamen) via the motor cortex^8^ or the hippocampus^9^.

The electrophysiological studies that have been published present a particularly confusing picture. Potegal et al.^10^ reported that electrical stimulation of the vestibular nerve evoked field potentials in the caudate nucleus of the anaesthetized cat, that could not be abolished by lesions of the vestibular cortical projection areas. Liedgren and Schwarz^11^ reported similar results in the anaesthetized squirrel monkey, with evoked potentials in the caudate nucleus and putamen in response to electrical stimulation of the vestibular nerve or vestibular nucleus. However, in contrast, the results of single neuron studies have been inconsistent. Segundo and Machne^12^ found that electrical stimulation of the vestibular labyrinth in curarized cats caused an increase in the firing rate of single neurons in the putamen and globus pallidus. On the other hand, Matsunami and Cohen^13^ reported that electrical stimulation of the contralateral vestibular nucleus in alert monkeys resulted in no change in the firing of neurons in the caudate nucleus, except at high currents that were sufficient to cause limb movement. In a more recent study, Rancz et al.^14^ electrically stimulated the superior vestibular nerve in anaesthetized rats and found both field potential and multi-unit responses in the striatum, corroborated by changes in fMRI activity. However, Stiles et al.^15^ conducted a systematic search for single neuron responses in the dorsomedial striatum to round window electrical stimulation in anaesthetized rats and found that only 6/507 neurons responded (1.1%) to the highest current amplitude (3 × the threshold for the generation of nystagmus). In general, there seems to be a lack of consistency between different studies, especially the effects of electrical stimulation of the peripheral vestibular system, on field potentials compared to single neuron responses in the striatum, although some of this may be due to differences in the levels of anaesthesia and/or species differences.

Despite the inconsistent electrophysiological evidence from electrical stimulation studies, a correlation has been reported between natural vestibular stimulation and activation of the striatum in freely moving mice^16,17^. PET and fMRI studies in humans have also shown responses in the putamen and caudate nucleus following either cold caloric vestibular stimulation or galvanic vestibular stimulation (GVS)^18–21^. In the most recent study, Qi et al.^22^ used focal electrical stimulation of the brain in epileptic patients and reported striking vestibular sensations with stimulation of the putamen and globus pallidus. In addition, there are a number of studies that have reported neurochemical changes in the striatum following peripheral vestibular lesions [e.g., Ref.^23^; see Ref.^24^ for a review]. Together, these data have helped fuel an interest in using GVS as a potential treatment for PD [see Ref.^24^].

One limitation of the previous electrophysiological studies is that they have focussed on the dorsomedial striatum, due to the view that sensory input is concentrated there^15^. However, recent studies have provided new insights into striatal organization^25^ and the tail of the striatum, unlike the dorsomedial striatum, has been reported to receive excitatory cortical projections from auditory, olfactory and somatosensory structures in the cortex and thalamus^26^. Interestingly, midbrain dopamine neurons that project to the tail of the striatum receive information from the substantia nigra pars reticulata, entopeduncular nucleus, zona incerta, globus pallidus, parasubthalamic and subthalamic nuclei^27^. This might suggest that neurons in the tail of the striatum receive a range of different kinds of sensory and motor information. The striatal tail has not previously been investigated in relation to vestibular input, and it is possible that, due to the concentration of other sensory inputs^25^, vestibular information may also be represented there. Therefore, in the present study, we investigated the effects of electrical stimulation of the peripheral vestibular system on local field potential responses in the striatal tail of anaesthetized rats.

## Materials and methods

### Animals

All experimental procedures reported here were approved by the University of Otago Animal Ethics Committee. Twenty-five adult male Wistar rats (8–9 weeks old) weighing between 280 and 350 g were used in the study. Animals were housed under a 12 h light–dark cycle in a temperature-controlled room (22 C ± 1 C) with free access to food and water. All methods were carried out in accordance with relevant guidelines and regulations. The study is reported in accordance with ARRIVE guidelines (https://arriveguidelines.org).

### Electrical stimulation of the round window

Animals were anaesthetised with 1.2 g/kg urethane (i.p., U2500; Sigma-Aldrich). During the surgery and recording, the subject's body temperature was monitored using a rectal probe (Harvard Apparatus) and maintained at 37 °C. The animal was placed into a custom-made nose bar, and the surgical procedure was performed under an ENT microscope (OPMI Pico, Zeiss, Hamburg, Germany). A retro-auricular surgical approach was used to expose the tympanic bulla, which was drilled open to approach the fenestra cochlea (round window). After cauterising the stapedial artery, a custom PFA-coated stainless steel bipolar stimulating electrode (coated diameter, 0.008 inches; A–M Systems.) was placed into the round window. In order to determine whether the stimulation was selective for the vestibular system and was independent of the auditory system, the cochlea was surgically lesioned in another 4 rats by carefully puncturing a hole at the apex and scraping the content with a fine needle. Although it is possible that the electrical stimulation could still have activated the auditory nerve directly, we reasoned that if auditory activation was implicated, we should at least have seen some decrease in the striatal responses following the cochlear lesions; in fact, even round window electrical stimulation has been reported to not affect the auditory cortex^28,29^.

The stimulating electrode was connected to an Isolator-11 stimulus isolation unit (Axon Instruments, Foster City, CA, USA) controlled by a Micro3 CED 1401 and Spike 2 software (Cambridge Electronic Design, Cambridge, England). In order to assess the correct placement of the stimulating electrode, vestibular nystagmus, an indication of vesibulo-ocular reflex activation, was visualised using video microscopy (Dino-Lite microscopic video camera). The threshold for the induction of nystagmus was determined by the lowest current that induced visible eye movement. Then the electrode was secured in place with dental cement. Based on previous studies^14,15,30^, a range of stimulation parameters was tested (square wave monophasic pulses at 100 and 333 Hz, 0.5, 1, 2 ms pulse length with 3 ms inter-pulse intervals; 2–10 monophasic pulses (a train) with a range of 10 s, 15 s, 20 s, at 30 s inter-train intervals, repeated 30, 35, and 50 times) in order to optimise the stimulation. It was found that only electrical stimulation trains consisting of 10 pulses of 1 ms monophasic square pulses with a 3 ms inter-pulse interval (333 Hz) delivered every 10 s for 50 times, could reliably activate vestibular nystagmus^14,15,30^. These stimulus parameters were used in all experiments. With the range of stimulation intensities used in this study, no obvious muscle movements (such as the neck, whisker or nose) were observed. This suggests that stimulation of the round window had very little or no effect on the facial nerve, and any effects on the auditory system were tested directly in the cochlea-lesioned animals. The experiments were not conducted in absolute darkness. However, the animals were deeply anaesthetized and the visual conditions were identical for each animal, i.e. a wall through a Faraday cage; therefore, we feel that it is very unlikely that visual stimulation contributed substantially to the responses.

### Eye movement recording

The vestibular nystagmus evoked by the electrical stimulation of the round window was visualised and recorded using video microscopy (Dino-Lite microscope video camera), which delivered uncompressed images with 30 fps at 3 MP (640 × 480 resolutions) and a 1.1 Aspect Ratio. Data from the video camera were displayed on a PC, and the recorded eye movements were analysed manually. Because we recorded eye movements only to confirm that nystagmus could be generated using the specific electrode placement, and to approximate its threshold, no attempt was made to quantify the eye movement velocity. However, the nystagmus could be seen very clearly and was usually torsional (see Supplementary Video File 1). Therefore, although we could not quantify the precise threshold velocity of the eye movements in response to the electrical stimulation, we could estimate the lowest intensity for triggering visible nystagmus. An example of a typical eye movement trace is shown in Supplementary Video File 1.

### Local field potential recording

After securing the stimulating electrode in place, the rat was transferred to the stereotaxic frame for implanting the local field potential (LFP) recording electrode in the tail of the striatum. A straight incision was made in the scalp to expose the skull, and the incision continued to the back of the animal's neck to insert a Ag/AgCl pellet reference electrode (Warner Instruments, Hamden, CT) under the skin. A single craniotomy was used with a dental drill (Foredom K1070) to expose the target area (AP: 2.28 mm, ML: 4.80 mm)^31^, either ipsilateral or contralateral to the stimulating electrode. A custom micro-LFP electrode (PFA coated stainless steel, coated diameter 0.008 inches; A-M Systems) was placed in a holder and stereotaxically implanted into the target area by using a scientific micromanipulator (IVM-3000, Scientifica, UK) under computer control (LinLab software, Scientifica, UK) at a rate of between 1 and 2 µm per sec. LFPs were recorded using a Micro3 CED 1401 system and Spike 2 software (Cambridge Electronic Design, Cambridge, England, version 7.10c × 86). The recording was performed at different depths in the tail of the striatum (DV: 5.5, 6 and 6.5 mm). The LFP electrode was connected to a headstage (NL100RK, Digitimer, Hertfordshire, UK). Fifteen min after implanting the LFP electrode the stimulation was started at the lowest current amplitude that evoked visible nystagmus. Then the intensity was increased by 50 µA until four times higher than the lowest intensity at each depth of the target area. Broadband LFP signals were digitised at 10,000 Hz after being amplified × 1000 by using an AC neurolog preamplifier (NL104A, Digitimer, Hertfordshire, UK) and low-pass filtered between 0 and 300 Hz using a dedicated AC-differential amplifier (NL104A, Digitimer, Hertfordshire, UK). A Grass audioamplifier (AM8, Indianapolis, IN) was used for audio feedback during recording. The LFP recording was configured so that it was triggered by the last pulse of the stimulation for all 50 trains of stimulation.

### Histology

Following the completion of the recordings, the brains were removed and fixed in 10% formalin for 48 h. The brains were then transferred to a phosphate buffer with 30% sucrose for cryoprotection and remained there until they had sunk. The brains were sliced into 50 μm coronal sections using a cryostat (Leica CM1950). Sections were stained with Cresyl violet and the electrode placement was visualised under a light microscope (Nikon Elipse, Ni-E).

The temporal bone histology was used to confirm the surgical lesions of the cochlea. Briefly, the temporal bones in both cochlea-lesioned and non-cochlea-lesioned rats were carefully dissected and transferred to 4% paraformaldehyde (PFA) in 10 mM phosphate-buffered saline (PBS) and incubated at room temperature for 2–20 h. Then the samples were transferred to 10% ethylenediaminetetraacetic acid (EDTA) for decalcification. The presence of spongey tissue indicated that decalcification was complete. The samples were then dehydrated through a series of graded ethanol solutions and xylene. The samples were then embedded in paraffin wax, cut into 4 μm sections, stained with haematoxylin and eosin and examined under a light microscope.

### Data analysis

All analyses were performed by using Spike2 software offline. The custom-written script was used to extract the last pulse train in order to obtain the LFP waveforms averaged across trials with respect to the stimulation trigger (500 single stimulations, 50 times for each average). Positive deflections in the LFP signal were classified as evoked potentials for additional analysis when larger than three times the SD in a 250 ms window preceding the stimulus. The amplitudes and the latencies of the peak positive deflections were averaged across rats at the same cortical depth. Then the stimulus intensity was normalised to the lowest intensity for triggering nystagmus for each animal for both the ipsilateral and contralateral responses. The data were first tested for the assumption of normality and then log transformed if necessary, in order to fulfil this assumption. Data were then analysed using Generalized Estimating Equations (GEEs) in SPSS 27, in order to take account of the correlation in the repeated measures^32^.

The datasets used and/or analysed during the current study are available from the corresponding author on reasonable request.

## Results

**Figure 1:**
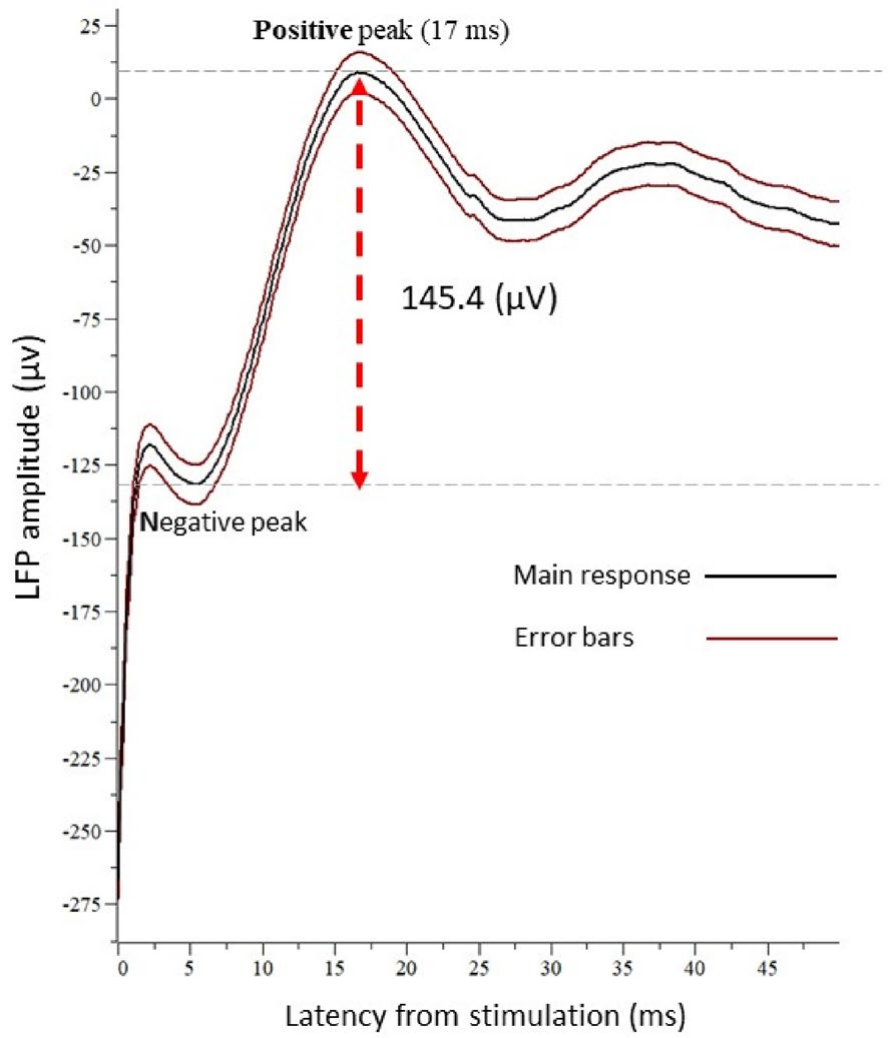
Evoked triphasic LFP response after electrical stimulation of the round window. The LFP recording was configured so that it was triggered by the last pulse of the stimulation for all 50 trains of stimulation. The trace represents an average of 50 sweeps ± 1 SE.

Electrical stimulation of the round window evoked a clear triphasic LFP, with a negative peak followed by a positive peak and then another negative peak (Fig. 1). In general, the positive peak of the waveform exhibited a latency of less than 22 ms.

**Figure 2:**
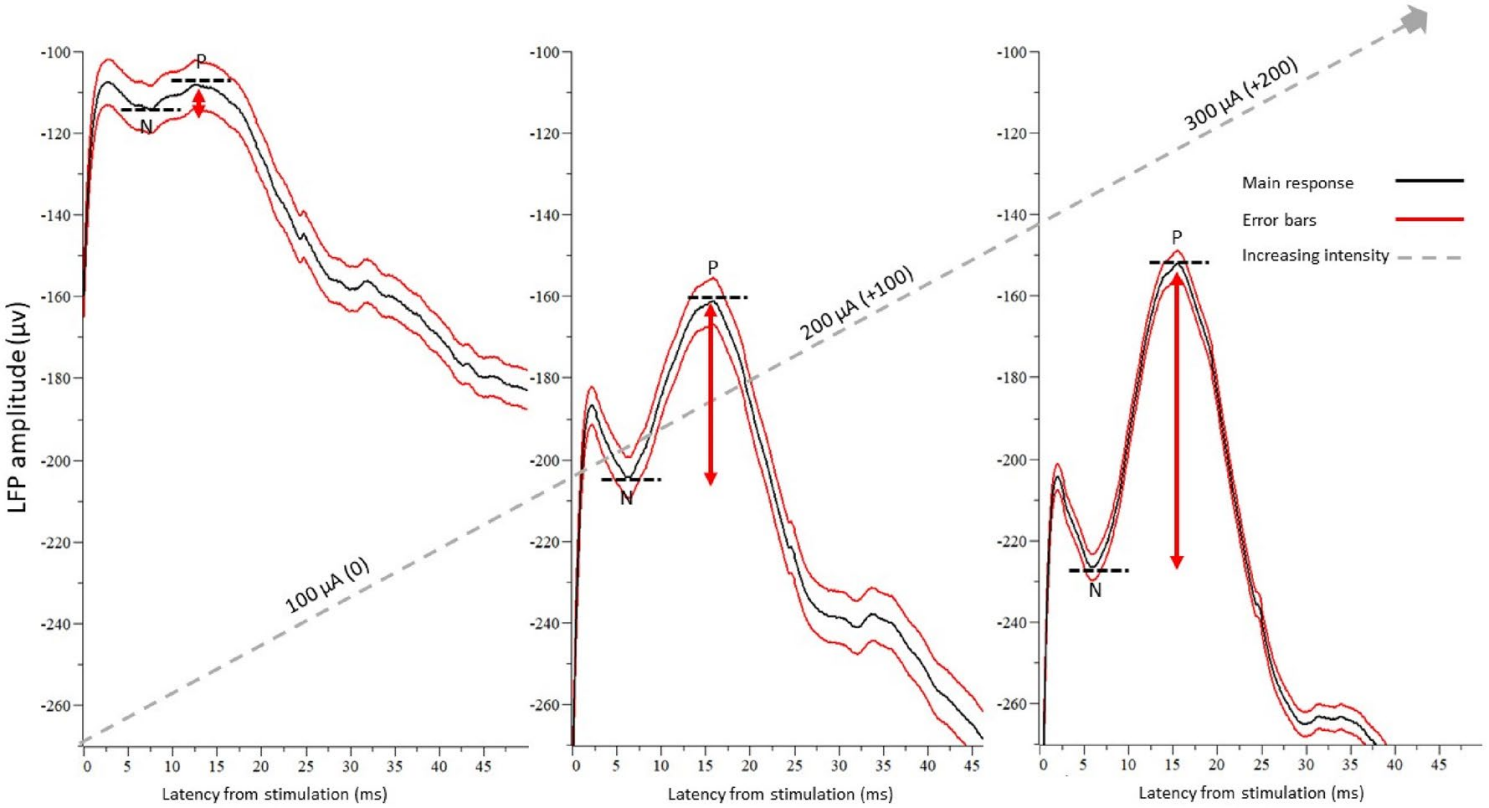
Example of the evoked LFP responses after increasing intensity of electrical stimulation. The LFP recording was configured so that it was triggered by the last pulse of the stimulation for all 50 trains of stimulation. The traces represent an average of 50 sweeps ± 1 SE.

As shown in Fig. 2, the amplitude of the responses increased as a function of stimulus intensity (F (1,4) = 69.19, P ≤ 0.0001; Fig. 2). while the latency of the response was almost the same even in the cochlea-lesioned rats.

**Figure 3:**
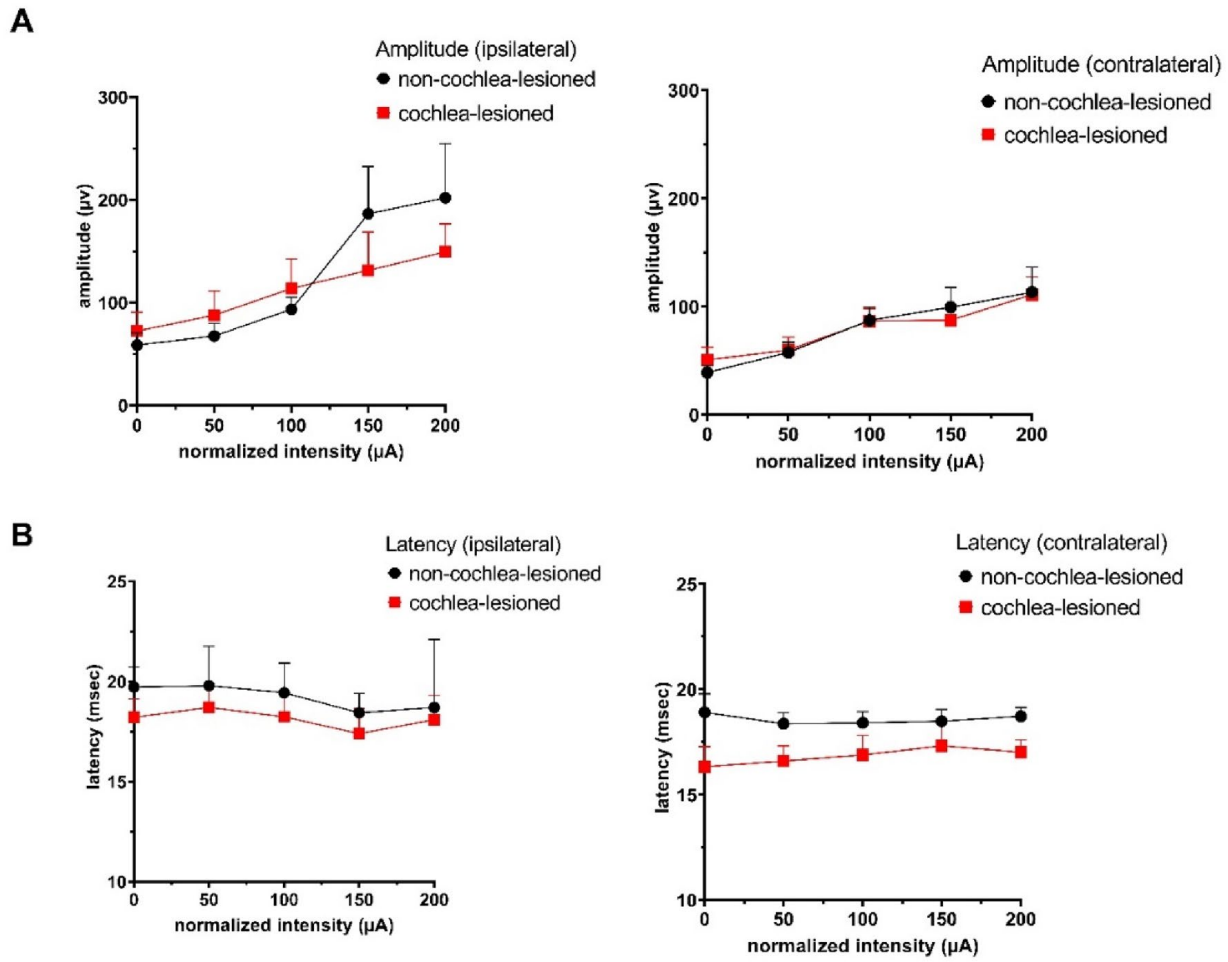
(**A**) Amplitude of the responses in the ipsilateral and contralateral tail of striatum in non-cochlea-lesioned and cochlea-lesioned rats evoked by stimulation of the round window. (**B**) Latency of responses in the in the ipsilateral and contralateral tail of striatum in non-cochlea-lesioned and cochlea-lesioned rats evoked by stimulation of the round window. Symbols represent means and bars 1 SE.

There were no significant differences between the responses in the striatum ipsilateral or contralateral to the stimulation. There were also no significant differences in amplitude between the non-cochlea-lesioned and cochlea-lesioned animals (Fig. 3A). There were no significant interactions amongst the factors.

In terms of the latency of the LFPs, there were no significant effects of current amplitude, side of recording or cochlear lesions, and no significant interactions (see Fig. 3B).

Analysis of the LFP amplitudes and latencies across different dorsoventral coordinates in the striatal tail revealed no significant differences (data not shown).

**Figure 4:**
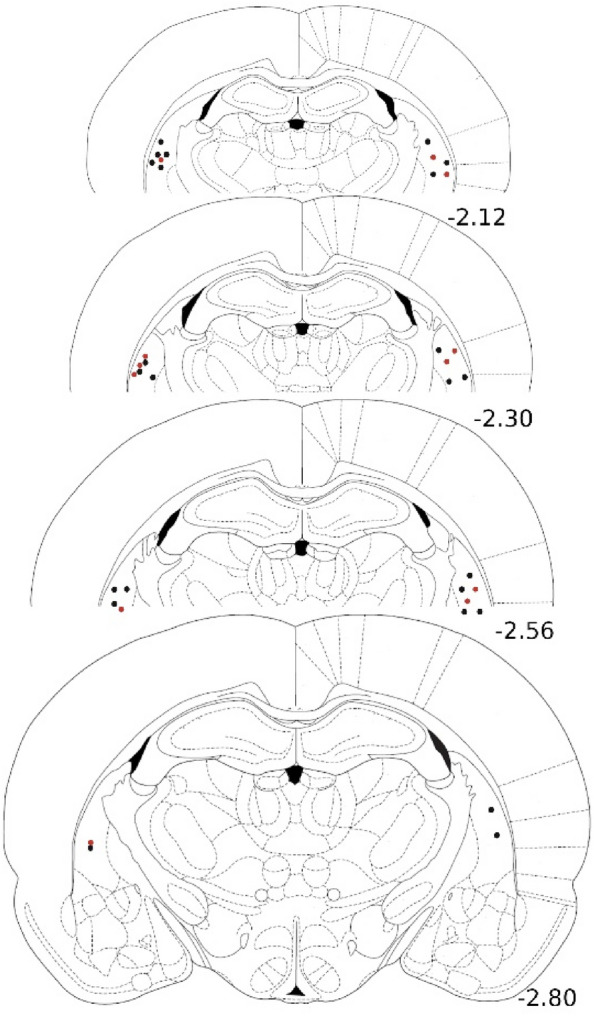
A schematic representation of the ipsilateral and contralateral electrode placements. The black and red dots represent the electrode placement in non-cochlea-lesioned and cochlea-lesioned rats, respectively.

**Figure 5:**
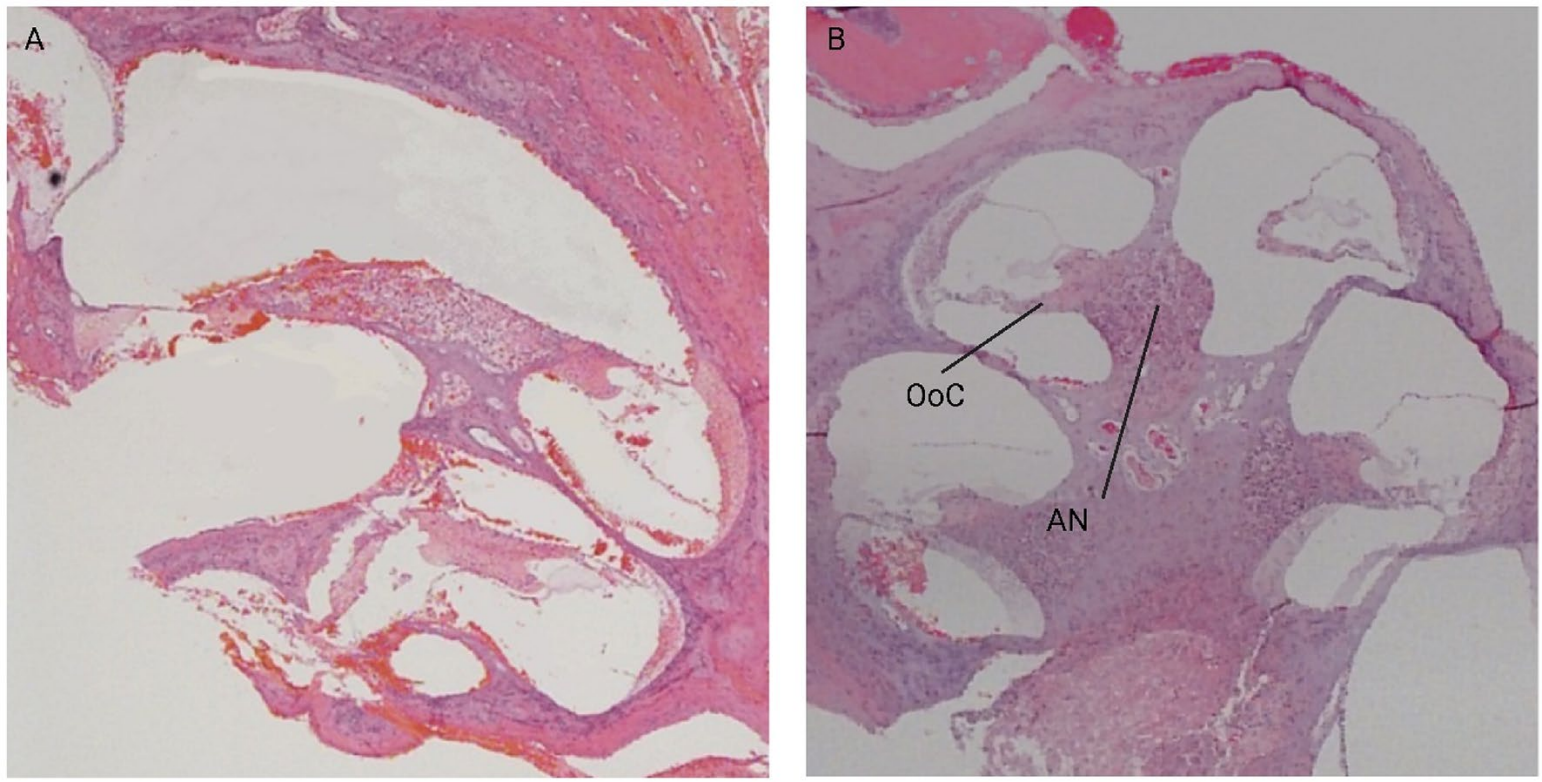
Histology of the cochlea-lesioned (**A**) and non-cochlea-lesioned (**B**) temporal bones, stained with haematoxylin and eosin showing the overall morphology. Auditory nerve (AN), the organ of Corti (OoC).

Histological analysis of the recording sites indicated that they were all located within the tail of the striatum (Fig. 4). Temporal bone histology indicated that for the group that received surgical lesions, these lesions were complete (Fig. 5).

## Discussion

Despite decades of speculation that the vestibular system transmits information about head movement to the striatum, the electrophysiological evidence supporting this idea is inconsistent and confusing [see Ref.^33^ for a review].

**Figure 6:**
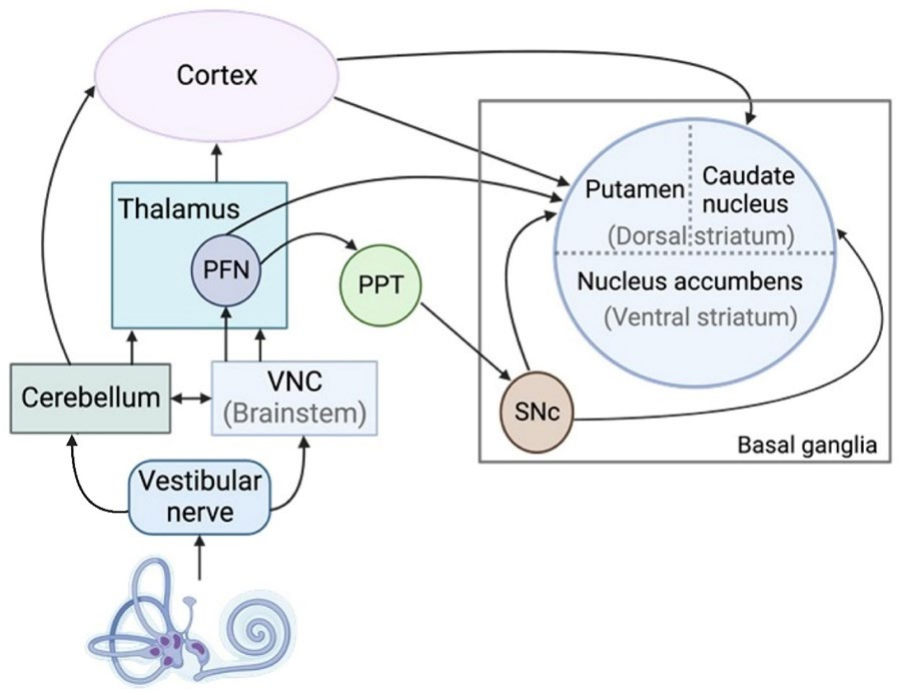
Possible neuronal pathways from the vestibular labyrinth to the striatum via the thalamus, cerebellum and cortex (adapted from Stiles and Smith^33^). *PFN* parafascicular nucleus, *PPT* pedunculopontine tegmental nucleus, *SNc* substantia nigra pars compacta. Created in BioRender.com.

On the one hand, human neuroimaging studies using PET and fMRI demonstrate responses in the putamen and caudate nucleus to cold caloric vestibular stimulation or GVS [e.g., Refs.^18–21^]. A recent study in humans showed that electrical stimulation of the putamen could evoke vivid vestibular sensations^22^. In fact, vestibular sensations were the second most frequent response category, accounting for 8 sites in 5 patients. One neurotracer study has provided evidence for a pathway between the medial vestibular nucleus and the dorsolateral putamen of the striatum via the parafasicular nucleus (PFN) [Ref.^7^; see Fig. 6]. On the other hand, the evidence for electrophysiological responses in the striatum to electrical stimulation of the vestibular system, is fragmented and discrepant. Several studies have reported field potentials in the caudate nucleus and/or the putamen in response to electrical stimulation of the vestibular nerve or vestibular nucleus^10,11,14^. Nonetheless, attempts to evoke single neuron responses in the striatum following electrical stimulation of the vestibular nerve have proven difficult and the results are inconsistent^12,13,15^. Of course, the inconsistencies between these studies are difficult to interpret because of species differences, the type of electrical stimulation used and whether it was applied to the peripheral vestibular system or the vestibular nucleus, the precise region(s) of the striatum (e.g. caudate nucleus versus putamen) from which recordings were made; and, particularly, whether anaesthesia was used, since anaesthesia can reduce spontaneous activity in the striatum significantly^34^.

The previous electrophysiological studies have tended to focus on the dorsomedial region of the striatum, since sensory input was believed to be concentrated there^15^. Recent studies, however, have provided evidence that the tail of the striatum, a relatively neglected area, receives excitatory projections from auditory, olfactory and somatosensory structures in the cortex and thalamus [Ref.^26^; see Ref.^25^ for a review]. In particular, neurons in the striatal tail receive projections from the substantia nigra pars reticulata, entopeduncular nucleus, zona incerta, globus pallidus, parasubthalamic and subthalamic nuclei^27^. To the best of our knowledge, no previous study has investigated whether the vestibular system projects to this area of the striatum. Given the concentration of other sensory inputs in this region^25^, vestibular information may also be represented there, and this was the motivation for the current study.

Since auditory information is represented in the striatal tail [Ref.^26^; see Ref.^25^ for a review], it was important for us to control for the possibility that the round window stimulation we used was not merely activating the cochlea. We did this by comparing LFPs in animals with surgical lesions of the cochlea and those without such lesions. We could find no statistically significant differences in the LFP amplitudes or latencies with or without cochlear lesions. This result, as well as the fact that LFPs could be evoked at relatively low amplitudes, suggested to us that the LFPs evoked by round window stimulation were due mainly to activation of the peripheral vestibular system. In fact, electrical stimulation of the round window has been reported not to activate single neurons in the auditory cortex or to evoke auditory sensations in humans^28,29^, so despite the proximity to the cochlea, round window stimulation does not necessarily result in cochlear stimulation.

In terms of potential current spread to the facial nerve, in addition to the absence of muscle twitching or whisker movement, it is important to note that the currents used in these studies were far lower than in our previous single neuron study conducted in the dorsomedial striatum^15^, where currents 3 × the nystagmus threshold (i.e., > 400 µA) were often necessary to evoke single cell responses. In that study^15^, as well as in the Rancz et al. study^14^, high current amplitudes were necessary in order to evoke neuronal responses, and in addition Stiles et al.^15^ found that the latencies were long, e.g. 50 ms on average, compared to the latencies in this study (< 22 ms), where LFP responses could be evoked at the threshold for nystagmus (see Fig. 3). Some previous studies have also failed to detect muscle twitching in guinea pigs following electrical stimulation of the round window^35^. Nonetheless, it is impossible to completely exclude the possibility that some low level of facial nerve activation occurred.

As might be expected, the amplitudes of the LFPs significantly increased with current intensity and intensity of nystagmus. Although the LFP amplitudes were slightly lower on the contralateral side, this was not significantly different, suggesting no laterality of vestibular input. Latency did not change significantly with the current amplitude or side. Interestingly, the lack of difference between the ipsilateral and contralateral striatum in terms of LFP amplitude and latency is consistent with the results of Rancz et al.^14^ and Stiles et al.^15^ in rats. This result suggests that vestibular input from one labyrinth is transmitted to both the ipsilateral and contralateral striatum. Given the different dynamic ranges of the left and right peripheral vestibular systems for angular and linear acceleration in a single direction, it is plausible that the striatum requires vestibular input from both sides in order to form a complete estimate of head movement^36^. However, these results are not consistent with the recent report of Qi et al.^22^, who found that vestibular sensations were evoked most easily from the right putamen. The issue of laterality needs to be studied further in both animals and humans.

The initial, positive-going peak of the LFP exhibited a relatively short latency of less than 22 ms. This is interesting because, in the study by Stiles et al.^15^, the sparse single neuron responses observed in the dorsomedial striatum, showed a latency of approximately 50 ms to round window stimulation. The shorter latencies of the LFPs observed in the current study suggest that the striatal tail responds differently to vestibular stimulation. Under urethane anaesthesia, these shorter latencies might be consistent with a subcortical pathway from the vestibular nucleus and/or cerebellum to the striatum via the PFN [Refs.^7,15^; see Fig. 6]. Although a lot of attention has been devoted to potential pathways from the vestibular nucleus to the striatum [e.g., Ref.^7^], it is important to remember that transcerebellar pathways are very likely, since the vestibular nerve projects monosynaptically to the ipsilateral cerebellum (see Ref.^37^ for a review; Fig. 6).

It is impossible to determine whether the responses observed in the striatal tail represent purely sensory information, or motor information, or both. Since the electrical stimulation evoked nystagmic eye movement, it follows that motor pathways were activated and part of the striatal responses may represent a copy of those motor commands or even feedback from motoneurons involved in the vestibulo-ocular reflexes, or both. Further studies, probably using single neuron recording, will be needed in order to address this issue.

Taken together, the results of this study indicate that sustained electrical stimulation of the peripheral vestibular system results in electrophysiological responses, in terms of LFPs, in the tail of the striatum, an area that has not been investigated previously. The responses were bilateral, with the amplitude increasing as a function of current intensity; however, current intensity had no significant effect on the LFP latency. Surgical lesions of the cochlea had no significant effects on LFP amplitude or latency, suggesting that the LFPs were due mainly to vestibular and not auditory input. These results suggest that the tail of the striatum may be an important site of integration for vestibular and other sensory inputs. It is possible that vestibular input to the striatal tail is part of the neurobiological basis of the effects of GVS on PD [see Ref.^24^ for a review].

## Supplementary Information

Supplementary Video 1.

Supplementary Information.
