# Supplementary material for: Vestibular modulation of the tail of the rat striatum
Source: Sci Rep. 2023 Mar 17;13:4443. doi: 10.1038/s41598-023-31289-1 (PMC10023713; doi:10.1038/s41598-023-31289-1)
Supplement: Supplementary file 2 — Supplementary Information. [file 41598_2023_31289_MOESM2_ESM.docx]

Fig. 1: A video example of an ipsilateral eye movement recording following stimulation of the round window
